# Modelling consumers’ choice of novel food

**DOI:** 10.1371/journal.pone.0290169

**Published:** 2023-08-28

**Authors:** Dawne Skinner, John Blake

**Affiliations:** Department of Industrial Engineering, Dalhousie University, Halifax, Nova Scotia, Canada; King Faisal University, SAUDI ARABIA

## Abstract

A variety of approaches to reducing the environmental impact of food production and consumption are being explored including technological solutions, such as food produced via biotechnological processes. However, the development of these technologies requires significant upfront investment and consumer acceptance is not guaranteed. The purpose of this research is to develop a system dynamics model to forecast demand, under multiple marketing and quality scenarios, for foods produced via novel technologies, using cellular agriculture as a case study. The model considers consumer heterogeneity, product awareness, word of mouth marketing (WOM), in-store marketing options, pricing options and product utility to estimate diffusion rates and market penetration. To our knowledge, there is no demand forecasting model available for food produced via novel technologies which relies on purchase intention data and incorporates all these factors. Therefore, this research closes a critical gap for that industry. Ultimately, the model shows that price and the consumers’ utility for the product drives the final demand regardless of marketing scenario. Further, the rate of diffusion was highest when product samples are provided in store for all scenarios except when product utility is low and the product price is high. Model results suggest that market saturation was reached within the 32-week trial period when the price of the cellular agriculture product was the same as a traditional product but not when the price was double that of traditional meat. Given the lack of available trial data, the model scenarios should be considered a *prior probability* which should be refined as more data becomes available.

## Introduction

As the environmental impacts of current food production processes have become better understood [1], there is significant interest in finding ways to improve environmental outcomes associated with the agri-food industry. Given the complexity of the global food supply chain and the diverse demands of consumers, a variety of approaches to reduce the environmental impact of food production and, perhaps, improve the resilience of food supply chains, have been explored. These include the promotion of lower impact diets, incentivizing a shift to more local and regenerative production processes and reducing food waste. Additionally, technological solutions, such as genetically modified crops, vertical agriculture, and biotechnological production processes, are currently being developed. However, consumer acceptance of these novel products and processes cannot be assumed.

Understanding the demand for products is critical for the economic viability of any company, as it reduces market uncertainty and strengthens the ability to make better decisions regarding product development, process capacity, pricing, and marketing strategies [2, 3]. While demand forecasting is an important undertaking in any industry, it is also known to be difficult [4] with low accuracy levels in general [5]. For products such as meat produced via cellular agriculture, that are not yet on the market, the accuracy of forecasting may be further hindered by a lack of pre-market trial data or the absence of a similar product against which to compare demand [3].

Further, many new product forecasting methodologies outlined in the literature do not account for low product awareness, the impact of word of mouth (WOM) or repeat purchases–all of which are important considerations for a novel fast-moving, perishable product. In the absence of data, traditional forecasting techniques have limited usefulness for predicting the demand for novel foods. To close this gap, a system dynamics model was developed that incorporates consumer purchase intention heterogeneity and awareness, product utility, word of mouth and in-store marketing as well as pricing strategies on diffusion and market penetration for a food product produced via a novel technology.

As a case study, the proposed model was used to develop a forecast for the uptake of the first meat product, assumed to be ground beef, produced via cellular agriculture in the US market. While several studies have been undertaken to determine consumer acceptance and purchase intention for these products, such as [6–9], to our knowledge, no trial demand modelling for these products has been presented in the academic literature or other publicly available source.

## Background

Accurate forecasting of demand for novel foods is complex, but critically important. Unreliable forecasts can reduce the economic and environmental viability of new products if a company relies on them to make investment and production decisions that result in over or under production [2–10]. Developing an appropriate model to forecast demand requires an understanding of consumer decision making around the adoption of novel foods [11] as well as an understanding of established forecasting techniques–including their limitations.

### Consumer decision making for novel food product adoption

Decision-making around engaging with a new food product in the pre-habitualization phase is governed by subjective “pros” and “cons” [12], primarily, a consumer’s expectations regarding product quality [13, 14]. This highlights the need to consider consumer heterogeneity in demand forecasting. In instances where no pre-market sales data is available, the heterogeneity of a consumer’s willingness to try or make an initial purchase of a novel product can be estimated using consumer purchase intention data [15, 16]. While studies have shown a positive correlation between purchase intention and behaviour [15, 16], purchase intention data should be interpreted cautiously for novel foods that are not yet on the market [17], since consumer intention is driven by taste and acceptability factors, which may be different after a product is introduced to the market [18]. For these reasons, forecasts for novel foods developed using purchase intention data or focus group data should be considered a *prior probability* which should be updated as more people become familiar with the product and pre-market test data becomes available [19].

It is known that a consumer’s repeat purchase probability relies on the extent to which a consumer’s quality expectations were met by their initial purchase [20]. While these expectations are primarily governed by taste, convenience, health/safety and comfortableness with the production methodology [13, 14], changing consumer preferences regarding product provenance, environmental footprint and ethical production, suggest that more consumers also consider the sustainability of a product in their purchase decisions [21, 22].

Product awareness is also assumed to be a critical component to consumer decision making. Therefore, if a consumer is unfamiliar with the product, its production process and the environmental (or other) benefits, it is unlikely that they would consider this product as a direct substitute for its traditional counterpart. Low consumer awareness is therefore believed to be a barrier to initial consumer demand.

**Fig 1:**
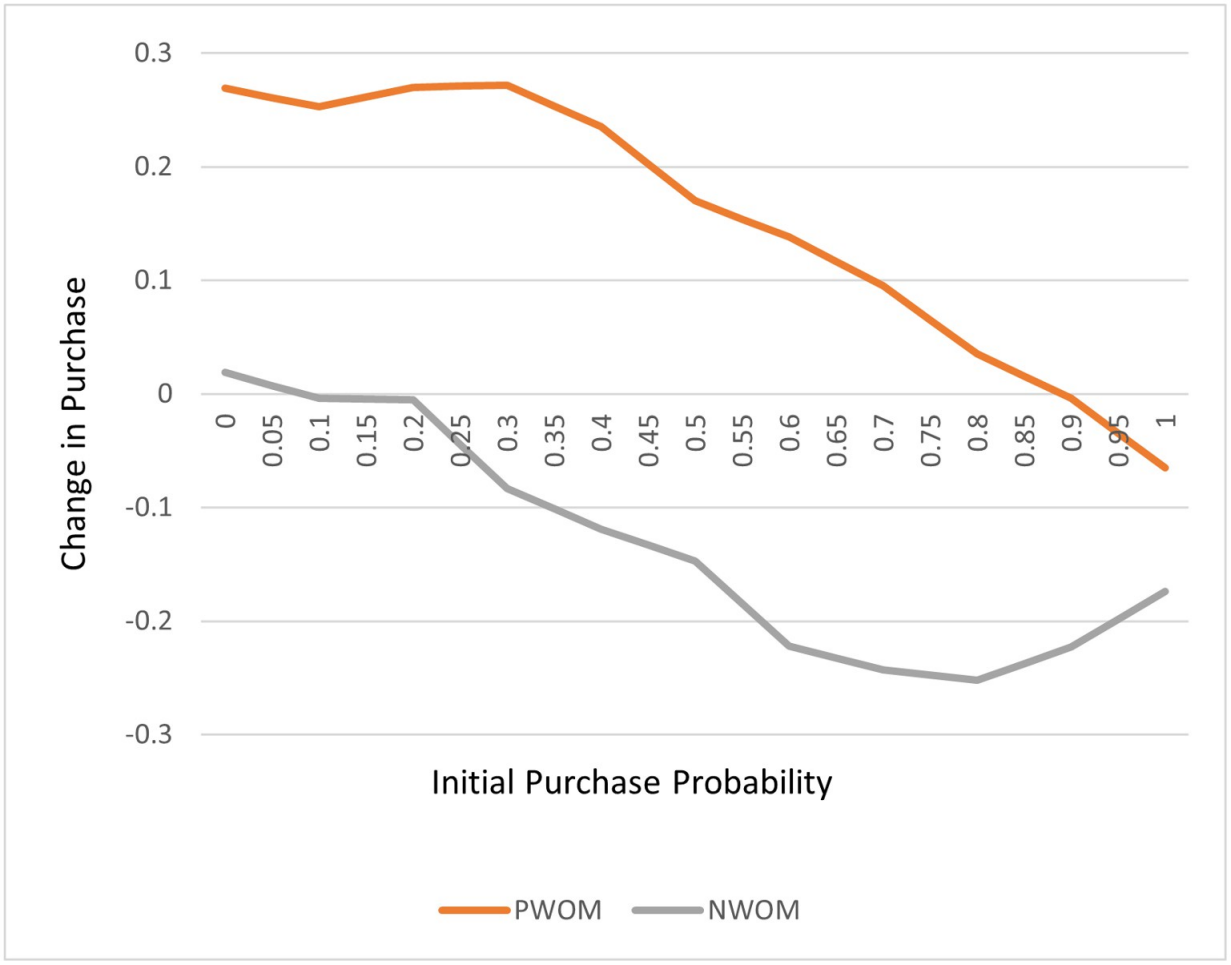
Absolute change in purchase probability with PWOM and NWOM (data from[24]) (in colour).

Increasing consumer awareness of new products and their benefits can occur via traditional marketing (advertising, in-store marketing, etc.) and through positive and negative word of mouth (PWOM or NWOM). WOM can also influence the purchase intention of a consumer who has not yet tried the product [23]. While several studies suggest that NWOM is more impactful on other consumers purchase intentions, a publication [24] summarizing the impact of WOM on purchase intention from 25 case studies, including food, consumer goods and restaurants, found that the impact of PWOM and NWOM was dependent on the receivers purchase probability, with PWOM having a bigger effect at lower initial purchase intentions and vice versa (Fig 1).

The impact of mixed WOM on the attraction of new customers has largely been ignored in the academic literature with most demand forecasting models focusing on positive WOM and advertising, all of which paints a product in a positive light [23]. This is a critical gap given the impact that NWOM has on purchase intention [23].

Most of the shoppers’ buying decisions are made inside of the stores, with two-thirds of supermarket purchase decisions being made while walking through the aisles [25]. This means that in-store marketing can be more effective than other advertising methods when it comes to influencing consumer purchase behaviours in the supermarket. Therefore, in-store marketing is expected to be the best point of engagement to influence consumer awareness and decision making regarding trying/buying a novel food. One study [25] found that in-store displays reached 72% of consumers’ attention while providing in-store samples was the most efficient method of getting consumers attention, reaching 88% of consumers’ attention.

Based on this, the forecast methodology used for novel food uptake should consider all these factors.

## Literature review

In the following section a brief summary of consumer choice modelling and cellular agriculture is presented.

### Consumer trial models

The accuracy of ight commonly used forecasting models (Table 1) for the trial component of new consumer packaged goods was compared and it was found that strictly concave forecasting models offered more accuracy than the classically assumed S-shaped diffusion models [2]. It was also shown that models which accommodate consumer heterogeneity outperform traditional diffusion models which assume a monolithic consumer group. Of the models investigated, they found that the most accurate new trial forecasting model was the exponential-gamma model, which assumes the time to trial for a randomly chosen consumer is distributed according to the exponential distribution and consumer heterogeneity is captured by assuming the purchase rate is distributed according to a gamma mixing distribution rather than a simple discrete distribution.

**Table 1:** Eight stochastic forecasting models for new consumer packaged goods reviewed in [2].

Exponential with ‘Never-Triers’	Weibell-gamma with ‘Never-Triers’
Exponential with ‘Never-Triers’ + Stretch Factor	Lognormal-lognormal
Exponential-Gamma	Double exponential
Exponential -Gamma with ‘Never Triers’	Bass Diffusion of Innovation

If used with a ‘never triers’ component, the exponential gamma model tends to underpredict demand, without one it tends to overpredict demand [2]. Limitations of this model include a lack of consideration of repeat purchases [2], the exclusion of WOM and marketing effects, and a failure to account for low product awareness, which is cited as a barrier to new product adoption [26].

### Combined trial plus repeat models

Logarithmic (or logistic) and Gompertz regression models have been used for new product trial and repeat demand forecasting. However, both models require the calculation of parameters that are difficult to estimate without trial data or without sales data for a similar mature product [17]. Another drawback of these models is a resultant S-shaped demand curve, which has been found to be less accurate than models resulting in concave demand curves [2].

The Awareness, Trial, Availability, and Repeat purchase (ATAR model) is used often in forecasting demand for consumer goods [5]. It relies on a decision-making hierarchy that only consumers who have awareness, willingness to try and have access to the product can make an initial purchase [5]. Repeat purchases can only be made after an initial purchase and will only be made by triers who have a higher utility for the product over other products [5]. The cumulative purchase volume for a product is estimated based on the number of initial and repeat purchases expected in a given period. One limitation of this model is that it requires some advance knowledge of the repeat purchase rate, which is not available for novel foods [5]. It also does not explicitly account for an increase in awareness through marketing and word of mouth.

### System dynamics modelling

System dynamics (SD) can be useful when modelling complex systems, including the diffusion and adoption of novel food products [27]. The benefit of SD modelling is that it enables the simulation of complex systems, driven by potentially complicated feedback relationships between variables and other system elements, to develop plausible scenarios even in situations where data may be limited [27–29]. However, to be an effective tool, the application of such an approach requires a robust understanding of the system under study.

### Cultured meat as a case study

Cellular agriculture is the production of animal agricultural products, including meat (also known as ‘cultured meat’), dairy, leather, etc., using biotechnology to grow animal cells and proteins in bioreactors rather than raising and slaughtering animals [30, 31]. While the industry is still in its early development, some studies suggest that this technology could significantly reduce the environmental impacts associated with animal agriculture [32, 33] while improving human health outcomes [30], food security [34] and animal welfare [31]. However, the cost to develop and scale these novel techniques and products can be prohibitive to their development [31]. Further, consumer acceptance of these products cannot be assumed. Understanding the potential demand for these products will enable companies to make informed decisions [2, 3] and provide the market intelligence needed to increase investment and the development of a dedicated supply chain [35].

## Proposed model

**Fig 2:**
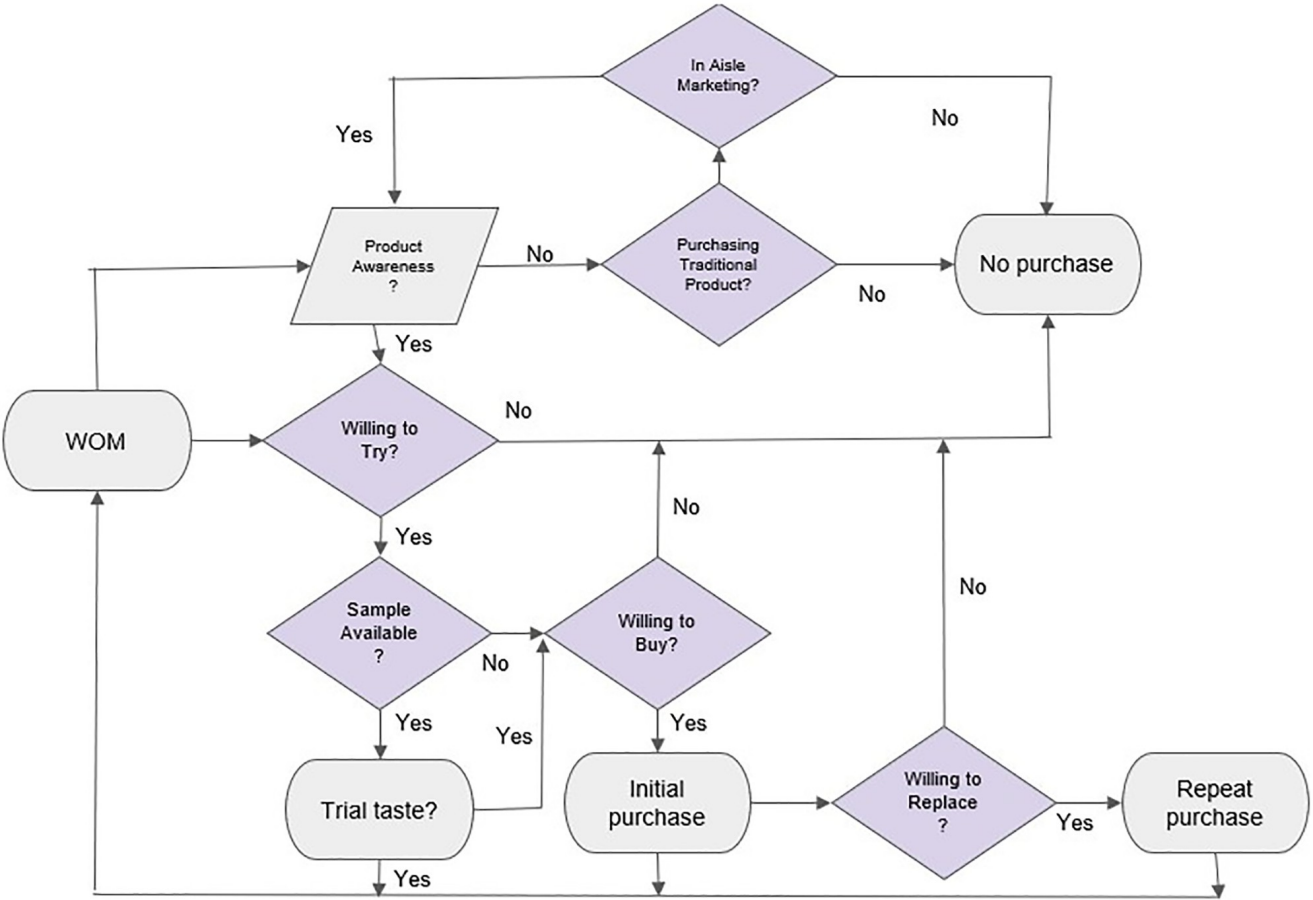
Proposed model of consumer decision making when choosing to try or buy a novel food product (in colour).

As previously discussed, consumer awareness, heterogeneity, product utility and the impact of marketing and word of mouth are all important factors in estimating consumers’ willingness to try, buy and make a repeat purchase of a novel food product. Given the complexity of the decision-making process and the unknowns related to product quality, marketing strategies and price, a system dynamic modelling approach was used to forecast diffusion and market penetration under various scenarios. Fig 2 summarizes the individual decision-making process when choosing to try, buy and make a repeat purchase of a novel food product at a retail store and the influence of in-store marketing (i.e. no marketing, manned kiosks with branded content or product samples) and WOM. Table 2 summarizes the model notation.

**Table 2:** Model notation.

*Symbol*	*Description*	*Assumptions*
*A*_*p*_ *(t)*	Consumer awareness of the product at time t	Variable, influenced by positive and negative WOM, in-store marketing, 0 ≤ *A*_*p*_(*t*) ≤ 1
*P*_*t*_ *(t)*	Consumers probability to try a sample (when available) at time t	Variable, based on pre-trial purchase intention data and influenced by positive and negative WOM, 0 ≤ *P*_*t*_(*t*) ≤ 1
*ω*	Percentage of consumers reached by in aisle marketing or sample availability in store	Parameter, based on level of marketing effort by company, 0 ≤ *ω* ≤ 1
*P*_*r*_ *(t)*	Consumers probability to make a repeat purchase at time t	Variable, influenced by a consumer’s utility for the novel product at price p, 0 ≤ *P*_*r*_(*t*) ≤ 1
*P*_*b*_ *(t)*	Consumers probability to make an initial purchase in the absence of samples at time t	Variable, influenced by consumers pre-trial utility for the product at price p, 0 ≤ *P*_*b*_(*t*) ≤ 1
*λ*	Purchase rate for the food category	Parameter, assumed to be the same as the demand for traditional product at price p
*Ω*	Positive WOM	Variable, effect is dependent on a consumer’s probability to try/buy as outlined in [23], 0 ≤ *Ω* ≤ 1
*Θ*	Negative WOM	Variable, effect is dependent on a consumer’s probability to try/buy as outlined in [23], 0 ≤ *Θ* ≤ 1
*α*	In- aisle marketing effectiveness	Parameter, effect is 0.72 as per[(24]
*μ*	Sample marketing effectiveness	Parameter, effect is 0.88 as per [24]
*U*	Estimate of consumer’s utility for the product	Parameter, dependent on product quality (taste, safety, convenience, etc) at price *p*. Assumed equivalent to the aggregate consumers probability of a repeat purchase.
*τ*	Fraction of consumers willing to make an initial purchase who do so during period t-1 to t	Exponential CDF, dependent on λ, 0 ≤ *τ* ≤ 1
*X(t)*	Cumulative number of initial triers or buyers at time *t*	Variable, dependent on consumer’s awareness, and probability to try/buy at price p
*I*_*b*_ *(t)*	Number of initial buyers during time period *t*	Variable, dependent on consumer’s awareness, and probability to buy at price p
*I*_*t*_ *(t)*	Number of initial triers during time period *t*	Variable, dependent on consumer’s awareness, probability to try and availability of samples
*R(t)*	Number of repeat buyers during time period *t*	Variable, dependent on consumer’s utility for the product at price *p*
S(t)	Total demand for novel product at time *t*	Variable, dependent on U, p, A_p_(t), I(t), P(t), *Ω*, *Θ*
V	Product Sample Size	Parameter, assumed to be 0.10 kg
γ	Strength of WOM	Parameter, assumed to be 0.151 as per [26]
*N*	Total population	Constant

**Note**: Product availability is assumed to be 100%.

### Model equations

#### If no marketing is done


$$A_{p}\left(t\right)=A_{p}\left(t-1\right)+Ib\left(t-1\right)/N+\gamma *\left[1-A_{p}\left(t-1\right)-Ib\left(t-1\right)/N\right]$$ (3.1)

$${\text{P}}_{\text{b}}\left(\text{t}\right)={\text{P}}_{\text{b}}\left(\text{t}-1\right)+\gamma *(\Omega *U\;-\Theta \left(1-U\right))*X\left(t-1\right)/N$$ (3.2)

$${\text{I}}_{\text{b}}\left(\text{t}\right)={\text{P}}_{\text{b}}\left(\text{t}\right)*A_{p}\left(t\right)*\tau *\;\left[N-X\left(t-1\right)\right]$$ (3.3)

R(t)=X(t−1)*U (3.4)

$$\text{S}\left(\text{t}\right)=\left[{\text{I}}_{\text{b}}\left(\text{t}\right)+\text{R}\left(\text{t}\right)\right]*\lambda$$ (3.5)

$$\text{X}\left(\text{t}\right)=\text{X}\left(\text{t}-1\right)+{\text{I}}_{\text{b}}\left(\text{t}\right)$$ (3.6)

#### If in aisle marketing is done


$$A_{p}\left(t\right)=A_{p}\left(t-1\right)+\left(\frac{Ib\left(t-1\right)}{N}\right)+\left(\alpha *\omega +\gamma \right)\left(1-A_{p}\left(t-1\right)-\frac{Ib\left(t-1\right)}{N}\right)$$ (3.7)

Eqs 3.2 to 3.6 apply

#### If in store samples are provided


$$A_{p}\left(t\right)\;=A_{p}\left(t-1\right)+\frac{Ib\left(t-1\right)}{N}+\frac{It\left(t-1\right)}{N}+\left(\gamma +\mu *\omega \right)*\left(1-A_{p}\left(t-1\right)-\frac{Ib\left(t-1\right)}{N}-\frac{It\left(t-1\right)}{N}\right)$$ (3.8)

$${\text{P}}_{\text{t}}\left(\text{t}\right)={\text{P}}_{\text{t}}\left(\text{t}-1\right)+\gamma *(\Omega *U–\Theta \left(1-U\right))*X\left(t-1\right)/N$$ (3.9)

$${\text{P}}_{\text{b}}\left(\text{t}\right)={\text{P}}_{\text{b}}\left(\text{t}-1\right)+\gamma *(\Omega *U–\Theta \left(1-U\right))*X\left(t-1\right)/N$$ (3.10)

$${\text{I}}_{\text{t}}\left(\text{t}\right)=[{\text{P}}_{\text{t}}\left(\text{t}\right)]*\tau *\;[N-X\left(t-1\right)]]*\omega$$ (3.11)

$${\text{I}}_{\text{b}}\left(\text{t}\right)=[{\text{P}}_{\text{b}}\left(\text{t}\right)*A_{p}\left(t\right)*\tau *\;\left[N-X\left(t-1\right)\right]]*\left(1-\omega \right)+\text{U}*{\text{I}}_{\text{t}}\left(\text{t}\right)$$ (3.12)

$$\text{R}\left(\text{t}\right)=\left[{\text{I}}_{\text{b}}\left(\text{t}\right)+\text{X}\left(\text{t}-1\right)\right]*\text{U}$$ (3.13)

$$\text{S}\left(\text{t}\right)={\text{I}}_{\text{t}}\left(\text{t}\right)\text{V}+\text{R}\left(\text{t}\right)*\lambda$$ (3.14)

$$\text{X}\left(\text{t}\right)=\;\text{X}\left(\text{t}-1\right)+{\text{I}}_{\text{b}}\left(\text{t}\right)+\left(1-\text{U}\right)*{\text{I}}_{\text{t}}\left(\text{t}\right)$$ (3.15)

#### Model description base case

Eqs 3.1–3.6 present the base case model, in which no marketing is done. Assume X(t) represents the cumulative number of persons to have made an initial purchase from time = 0 to time = t. Assume also, that A_p_(t) is the fraction of the population that is aware of the product at t. If N is the total population and γ is the strength of WOM (0 ≤ γ ≤ 1), the fraction of the population in period t that is aware of the product A_p_(t) is A_p_(t-1), the fraction of the population aware in the previous period, plus the fraction of the population that has made an initial purchase of the product I_b_(t-1)/N and the fraction of the unaware population (1-A_p_(t-1)- I(t-1)/N) multiplied by the product of γ. This relationship is shown in Eq 3.1.

Eq 3.2 defines the proportion of the population willing to make a purchase of the product at time t, P_b_(t). It equals the proportion willing to buy in the previous period P_b_(t-1) plus changes in intention due to positive WOM, (Ω*U), less changes due to negative WOM, Θ(1-U), multiplied by the fraction of the population that has tried the product, X(t-1)/N, and the strength of WOM, γ.

Eq 3.3 defines the fraction of the population in period t willing to make an initial purchase I_b_(t). It is assumed that there is a lag between purchase intent and actually purchasing the product and that this lag (τ) is exponentially distributed with a mean of rate of 1/λ. Thus, τ is the fraction of people willing to purchase the product that will purchase in this period and is derived from the cumulative distribution function of an exponential distribution with mean τ. For instance, if λ = 5 and τ = 0.2, then approximately 18% of the population of persons willing to buy will purchase in any given week. In total, the number of persons making a purchase in period t is equal to the proportion of the population that are willing to purchase P_b_(t) multiplied by τ, A_p_(t) and the number of persons who have not tried the product [N − X(t- 1)]

Eq 3.4 returns the number of repeat buyers in period t, R(t). This number is equal to the number of persons to have tried the product in period t– 1, X(t-1), multiplied by the aggregate consumer utility for the product U.

S(t), defined in Eq 3.5, calculates the volume of product sold in period t. It is the sum of the number of initial buyers, I_b_(t), from Eq 3.3 plus the number of repeat buyers, R(t), from Eq 3.4 multiplied by λ which is the purchase rate per time period t.

Finally, Eq 3.6 updates the number of persons who have tried the product in period t, X(t). This is the sum of the number of persons in the previous period, X(t-1) plus the number of persons to have tried the product in period t, I_b_(t).

### Model description with in-aisle marketing

If in-aisle marketing is included as a diffusion strategy, there is a positive influence on consumer awareness of the product, and it is expected that diffusion will be more rapid. To model this situation, Eq 3.1, which defines awareness in period t, A_p_(t) is updated to be the fraction of the unaware population (1-A_p_(t-1)–I_b_(t)/N) multiplied by the product of the sum of γ and an additional term α representing the influence of aisle marketing multiplied by the percentage of consumers who have been exposed to the in-aisle marketing (ω). Eqs 3.2 to 3.6 carry over from the base case.

### Model description with store samples

Eqs 3.8 to 3.15 model the situation where consumers are provided with in-store samples as a diffusion strategy. Again, if samples are provided, it is expected that the rate of diffusion will be increased over both the base case and the in-aisle marketing scenario. Eq 3.8 defines A_p_(t), consumer awareness in period t similarly to Eq 3.7 with the addition of I_t_(t-1), to account for the number of people sampling the product in store, as well as the substitution of μ, the sampling marketing effectiveness, for α, the aisle marketing effectiveness.

Eqs 3.9 and 3.12 mirror Eqs 3.2 to 3.6 in the base case scenario with the addition of P_t_(t) and I_t_(t) as the probability of trying a sample and the number of triers (samplers) in period t. The equation for I_b_(t) is modified to include the proportion of consumers who have sampled the product in period t and made a purchase in the same time period. Eq 3.14 is similar to Eq 3.5 in the base case with the addition of the mass of samples (V) consumed as part of the total demand.

## Understanding consumer willingness to try/buy cultured meat

Several studies have been conducted in various geographies to determine consumers’ awareness of, and willingness to try or make an initial purchase of cultured meat, such as [6–9]. The results of these surveys suggest that an individuals’ willingness to engage with these products is highly influenced by their familiarity with the product and production process [6, 7, 9, 36–38], their utility for the differentiating features of these products [39–42] and individual characteristics including food neophobia [7, 40, 43–47], gender [7, 43, 48, 49], age [43], political beliefs [7] and religious beliefs [50]. The key takeaway from this is that any model used to forecast initial demand for a cultured meat product must consider consumer awareness, consumer heterogeneity and product utility.

### Estimate of initial demand for cultured ground beef product in US

Only one source of publicly available raw purchase intention data (available in the Supplementary Materials of [7]) was identified in the literature. The study surveyed over 600 US citizens on their awareness of, willingness to try, buy and replace traditional meat with cultured meat [7]. The respondent population was biased towards politically left-leaning respondents, which they found were more likely to engage with the product, however the authors concluded that the data was still broadly representative of the population. This appears to have been confirmed by a more recent study. [51] which had similar survey results.

The survey utilized a Likert-scale from 1 (absolutely) to 5 (never) to indicate a respondent’s willingness to try, buy and replace [7]. To model the willingness of US consumers to engage with the product, this survey data was transformed to a weighted purchase probability as described by [26] (See Table 3 and Eq 3.16).

$$\text{Pr}\left(i\right)=\;\Sigma w\left(i\right)*\left(\frac{n\left(i\right)}{N}\right),\;i=1,2\ldots 5$$ (3.16)

Where N = total number of respondents, n(i) is the number of respondents choosing a specific category and w(i) is the weight subscribed to each response category. The population awareness was calculated as the proportion of respondents who answered that they definitely or probably knew what cultured meat is. The outcome of these calculations suggest that A_p_(0) = 0.30, P_t_(0) = 0.66, P_b_(0) = 0.46 and P_r_(t) (i.e. U) = 0.44.

**Table 3:** Purchase probability.

Likert Scale	Probability
1 (Definitely will try/buy/repeat)	1
2 (Probably will try/buy/repeat)	0.75
3 (May/may not try/buy/repeat)	0.5
4 (Probability will not try/buy/repeat)	0.25
5 (Definitely will not try/buy/repeat)	0

Modeling was conducted at two prices (one at price parity with traditional ground beef (2021 USD), the other at double that price) to determine the potential impact of price on demand. The survey data from [7] suggests that consumers are willing to pay the same as, or slightly less for, cultured beef compared with traditional beef. Therefore, we assume that the price elasticity of demand for cultured beef is similar to that of traditional beef. We assume, further, that consumers do not substitute other traditional meat sources (chicken or pork) for ground beef if the price of a cultured meat substitute exceeds that of traditional ground beef. In 2021, the average retail price for ground beef was US$9.39/kg [52] and the purchase rate was 0.225 kg per week per capita [53]. The estimated price elasticity of demand suggests that a 1% increase in the price decreases the quantity of beef consumed in the US by 2.33% [54]. Assuming a negative exponential price elasticity of demand, a 100% increase in price would yield a purchase rate of approximately 0.044 kg/week/capita.

As there is little information regarding the quality characteristics of these products, product utility was modeled as high (0.8), medium (0.44) and low (0.2). Respondents in [7] indicated that their main reason for being unwilling to engage with cultured meat was their presumption that it would not taste good or that it would be otherwise unappealing (79% of respondents). Therefore, if cultured meat can exceed taste expectations, it is anticipated that this would translate to a higher willingness to replace traditional meat with cultured meat. For the scenarios where marketing initiatives were included, it was assumed that 25% of consumers were exposed to those initiatives.

## Results

**Fig 3:**
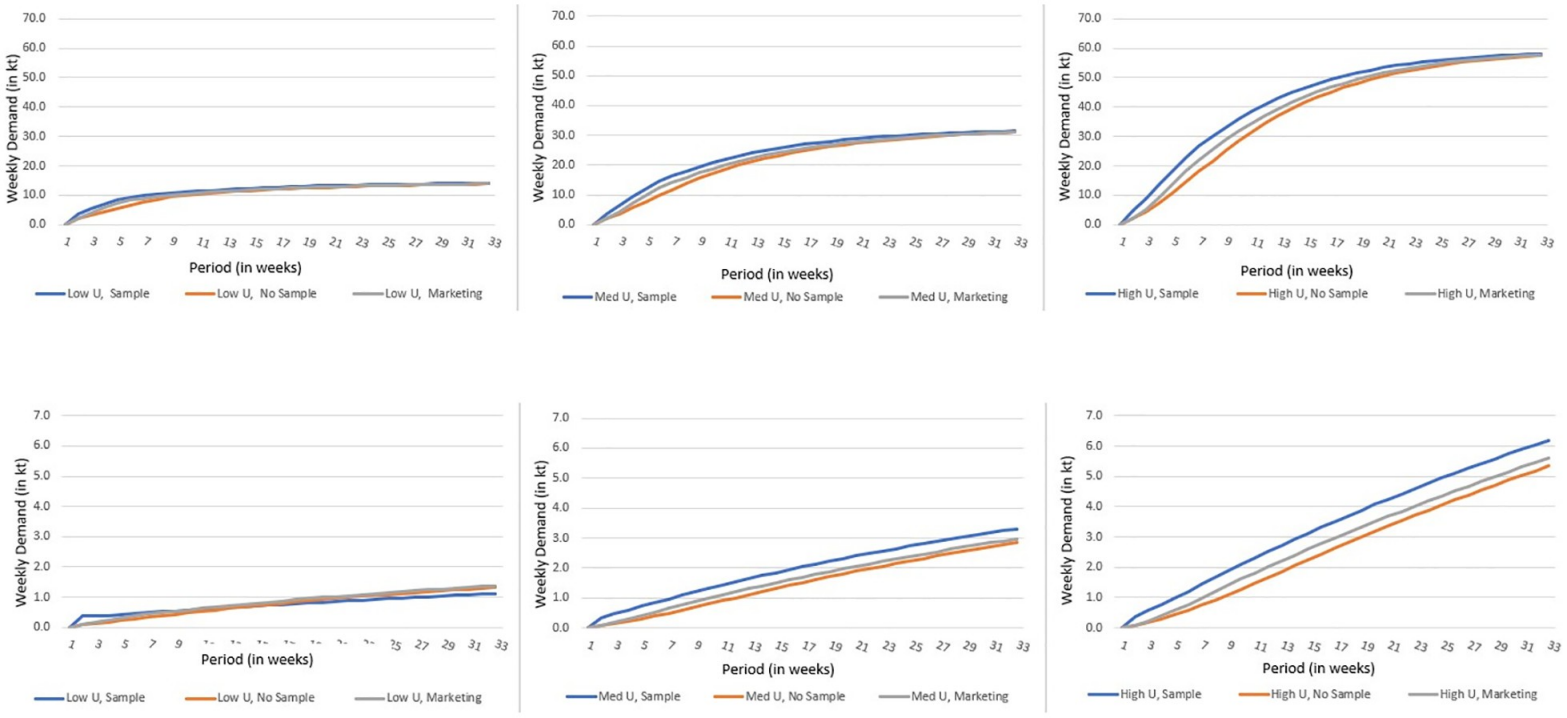
**a to c**: Forecasting demand for cellular agriculture derived ground beef under various quality and marketing assumptions and a low price (in colour). **d to f**: Forecasting demand for cellular agriculture derived ground beef under various quality and marketing assumptions and a high price (in colour).

Results of the modeling are shown in the figures below. Fig 3a to 3f present model results for product demand (in kt per week) during the 32-week trial period under the three different marketing strategies (WOM only, Sample, and In-Aisle Marketing) and different values of consumer utility for the product (Low, Medium, and High). Fig 3a–3c provides model results for cultured meat at price parity with traditional ground beef while Fig 3d–3f model demand for cultured meat when it is double the price of traditionally produced ground beef.

As anticipated, for the trial period, product demand is higher at the lower price (Fig 3a–3c), compared to the higher price (Fig 3d–3f), regardless of marketing strategy or product utility. Not surprisingly, relying only on word-of-mouth marketing yielded marginally slower diffusion and adoption compared to providing samples and in aisle marketing for all scenarios except when the product utility was low, and the price was high (Fig 3a). Under this scenario, providing samples was found to be counterproductive.

**Fig 4:**
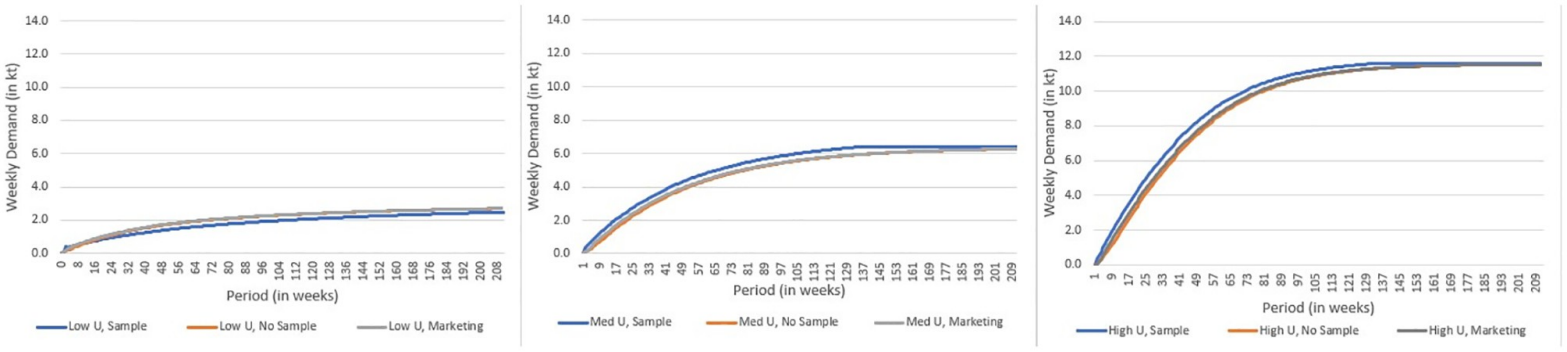
**a to c**: Forecasting demand for cellular agriculture derived ground beef under various quality and marketing assumptions and a high price until steady state demand is reached (in colour).

While this suggests that some form of marketing is advantageous for most scenarios, when steady state is reached (at the end of the trial period at price parity (Fig 3d–3f) and later when the price is high (Fig 4a to 4c)), the final weekly demand is similar regardless of marketing strategy deployed. This aligns with the findings of others who determined that decision-making around engaging with a new food product is driven by product quality expectations [12] and repeat purchase probability is driven to the extent to which these expectations are met [20]. As expected, the product with high utility and low price resulted in the highest demand.

Data for the model runs is available in S1 and S2 Tables.

## Conclusions

A system dynamics model was developed to estimate demand for a 32-week trial period for the first cultured meat product (assumed here to be ground beef) introduced to the US market. Model simulations using two price points, three marketing scenarios and three values for average product utility were run to identify the impact on diffusion and market share. In the absence of trial data, available purchase intention data was adjusted and used to estimate consumer heterogeneity and traditional meat purchase rates were used to estimate purchase rate and time to trial.

As expected, price dictates the speed of diffusion and adoption. Additionally, the product price and customer utility dominate the value of any marketing scheme. Further, while providing samples increased product diffusion for most scenarios, in the instance when the product is pricy and the quality (taste) is bad, providing samples resulted in lower overall adoption suggesting that giving consumers an opportunity to judge the product before purchasing can reduce adoption as might be expected. Choice of marketing strategy only marginally impacted product diffusion and did not impact product demand once market saturation was achieved.

It should be noted that the predicted speed of diffusion and adoption of a cultured meat product will vary according to the data used. The data used in this model is based on available survey responses from consumers who have not yet tried the product and therefore actual values of parameters such as consumer utility are unknown. Thus, the actual amount of product sold during a trial period could be different than estimated in this paper; however, the trends for diffusion described in this paper will hold regardless of the actual value. This highlights the benefit of our approach. While there are multiple and well-known forecasting models available with which to forecast product demand, none have been identified that consider the variety of factors that influence consumer decision making around trying and buying novel foods. While the value proposition of novel foods and food production processes -such as cellular agriculture- is enticing, several studies have found that a consumer’s willingness to engage with these products varies. Understanding potential demand for these products is critical to helping companies working on novel food production processes to make pricing and production capacity decisions.

## Supporting information

S1 Table Weekly demand during a 32-week trial period for cellular agriculture derived ground beef under various quality and marketing assumptions and a low price. (XLSX)

S2 Table Weekly demand during a 32-week trial period for cellular agriculture derived ground beef under various quality and marketing assumptions and a high price. (XLSX)
